# The expression of *Gli3*, regulated by HOXD13, may play a role in idiopathic congenital talipes equinovarus

**DOI:** 10.1186/1471-2474-10-142

**Published:** 2009-11-19

**Authors:** DongHua Cao, ChunLian Jin, MeiHong Ren, ChangKun Lin, Xuan Zhang, Ning Zhao

**Affiliations:** 1 Department of Medical Genetics, China Medical University, Shenyang, 110001, PR China; 2 Department of Test, No.202 hospital of People's Liberation Army, Shenyang, 110003, PR China

## Abstract

**Background:**

Idiopathic congenital talipes equinovarus (ICTEV) is a congenital limb deformity. Based on extended transmission disequilibrium testing, *Gli-Kruppel family member 3 *(*Gli3*) has been identified as a candidate gene for ICTEV. Here, we verify the role of *Gli3 *in ICTEV development.

**Methods:**

Using the rat ICTEV model, we analyzed the differences in *Gli3 *expression levels between model rats and normal control rats. We used luciferase reporter gene assays and ChIP/EMSA assays to analyze the regulatory elements of *Gli3*.

**Results:**

*Gli3 *showed higher expression levels in ICTEV model rats compared to controls (P < 0.05). We identified repressor and activator regions in the rat *Gli3 *promoter. The *Gli3 *promoter also contains two putative Hoxd13 binding sites. Using EMSA, the Hoxd13 binding site 2 was found to directly interact with Hoxd13 *in vitro*. ChIP assays of the Hoxd13-*Gli3 *promoter complex from a developing limb confirmed that endogenous Hoxd13 interacts with this region *in vivo*.

**Conclusion:**

Our findings suggest that *HoxD13 *directly interacts with the promoter of *Gli3*. The increase of *Gli3 *expression in ICTEV model animal might result from the low expression of *HoxD13*.

## Background

Idiopathic congenital talipes equinovarus (ICTEV) is a congenital limb deformity that affects 0.3-7% of live births worldwide [1]. It is defined by fixation of the foot in cavus, adductus, varus and equinus (inclined inwards, axially rotated outwards and pointing downwards) with related soft tissue abnormalities [2]. The mechanism underlying the development of ICTEV remains unclear, and neurological, muscular, bony, connective tissue and vascular mechanisms have all been proposed as contributing factors [2-5]. Although some studies have suggested that both genetic and environmental factors lead to ICTEV, twin studies, ethnic studies and pedigree analyses suggest a genetic basis for the condition [6]. Furthermore, studies have suggested that a significant ICTEV risk can be attributed to unknown disorder-causing genes [1]. Overall, little is known about the pathogenesis of human ICTEV.

Many candidate genes for this disorder have been proposed because the molecular and cellular components of vertebrate limb bud development are well known. Specialized regions of the developing limb bud, such as the zone of polarizing activity (ZPA), the apical ectodermal ridge (AER) and the non-ridge ectoderm, direct and coordinate the development of the limb bud along the anterior-posterior (AP), dorsal-ventral (DV) and proximal-distal (PD) axes in a pattern conserved for tetrapods [7]. Distal limb development along the AP (thumb to little finger) axis is governed primarily by the ZPA. The major signaling molecule with polarizing potential in the ZPA is Sonic hedgehog (Shh) [8], which plays a central role in pattern formation in the embryo [9] and is a key signal in establishing different digit fates along the AP axis of the vertebrate limb bud [10]. In *Drosophila*, hedgehog (Hh) signaling is mediated by the Cubitus interruptus (Ci) protein, a zinc finger transcription factor. In birds and mammals, Ci homologs constitute the three member *Gli *family (*Gli1, Gli2, and Gli3*). All three *Gli *genes are expressed in the developing limb, but only *Gli3*, a direct intracellular mediator of Shh [11-13], is necessary for limb patterning [14,15]. With a C-terminal repressor region and an N-terminal activator region, *Gli3 *is a bipotential transcription factor that can activate or repress some of the same target genes [16]. During vertebrate limb development, Shh signaling prevents the processing of the full-length Gli3 (Gli3-190) to a short form (Gli3-83) that functions as a strong repressor. In both mouse and chick limb buds, the repressor form of Gli3 is present in an anterior-posterior gradient with the highest levels in the anterior part of the limb bud where Shh signaling is at its lowest levels. The genetic data of the Shh, Gli3 and double-compound mutants indicate that the phenotype in the absence of Shh is caused by an excess in the Gli3R form that suppresses gene expression, cell survival and distal progression of limb bud development. Gli3 and Shh reciprocally restrict each other to control the normal limb morphogenesis [17].

A third family of transcription factors involved in limb development is the *HOX *family, which is evolutionarily conserved and plays a fundamental role in patterning the AP axis of developing embryos. Each HOX protein mediates cellular events during limb morphogenesis [18,19]. The physical position of the *Hox *gene within each cluster corresponds closely to their temporal and spatial expression patterns during development. Thus, genes at the 3' end of the clusters, such as *HOXD1*, are expressed early in the anterior and proximal regions, whereas genes at the 5' end, such as *HOXD13*, are expressed later in posterior and distal regions [20]. In early limb bud development, the *Gli3 *anterior expression overlaps the *HoxD *posterior expression, indicating that a genetic interaction between a 5' HoxD member and *Gli3 *regulates digit formation [21]. Biochemical and transfection analyses provide support for the physical interaction of the 5' HoxD protein and Gli3 protein via the homeodomain [21]. This interaction can convert the truncated Gli3 repressor form into an activator of its target promoters [21].

Most *Gli3 *research focuses on digit abnormalities and deformities. *Gli3 *mutations cause limb development disorders, such as Greig cephalopolysyndactyly syndrome (GCPS) [22], Pallister-Hall syndrome (PHS) [23], postaxial polydactyly type A (PAP-A) [24] and preaxial polydactyly type IV [25]. Association analyses suggest that *Gli3 *and *HoxD13 *are associated with ICTEV [26-28]. To confirm a role for *Gli3 *in ICTEV, we analyzed *Gli3 *expression in ICTEV model rat embryos. To understand how a change in the expression of *Gli3 *affects ICTEV, we investigated the interaction between *Gli3 *and HoxD13.

## Methods

### ICTEV patients and normal controls

Flexor hallucis longus was from 20 ICTEV patients (13 men and 7 women) aged 4-12 years (mean 6.7 years) and peripheral blood was from 84 ICTEV patients (50 men and 34 women) aged 3-12 years (mean 6.2 years). All patients were recruited from the Department of Pediatric Orthopedic Surgery, Second Affiliated Hospital of China Medical University. Informed consent was obtained from the patients and the study was approved by the China Medical University Ethics Committee. The probands showed the typical ICTEV phenotype (a fixation of the foot in adduction, supination and varus). Flexor hallucis longus and lung tissue were from nine normal cadavers (5 men and 4 women) aged 5-11 years (mean 7.5 years) as controls.

### A rat ICTEV model

Forty pregnant Wistar rats were obtained from the experimental animal center of our university. The ICTEV phenotype in rat embryos was induced by administration of 135 mg/kg all-trans-retinoic acid (ATRA) on gestation day 10 (GD10), as previously described [29,30]. All studies were performed with the approval of the experimental animal committee at our university (SCXK 2008-0005).

### RNA isolation and expression analysis

The *Gli3 *gene is expressed as an 8.5 kb mRNA in tissues such as the postnatal testis, myometrium, placenta and lung [31]. We investigated whether the gene was also expressed in the hindlimb. RNA was therefore extracted from the flexor hallucis longus of ICTEV patients and normal controls using a Tissue RNA kit (Tiangen, China) according to the manufacturer's protocol. RNA was also extracted from lung tissues of normal cadavers as a positive control. Using a reverse transcription kit (Promega, USA) according to the manufacturer's instructions, cDNA was generated. For *Gli3*, the forward and reverse primers were 5'-TTT TCC AAC ACA GAG GCC TAT TC-3' and 5'-ATC TTG GAC CTC TTG TTG TGC AT-3', respectively. Human β-actin forward and reverse primers were 5'-TCA CCC ACA CTG TGC CCA TCT ACG A-3' and 5'-CAG CGG AAC CGC TCA TTG CCA ATG G-3', respectively. PCR reactions contained 40 ng cDNA, 1.2 mM MgCl_2_, 20 mM dNTPs, 320 nM of each primer and 2U Taq polymerase in a 25 μl total volume. PCR reactions had an initial denaturing step at 94°C for 5 minutes, followed by 35 cycles of denaturing at 94°C for 45 sec, annealing at 58°C for 45 sec and extension at 72°C for 60 sec, and ended with a final extension at 72°C for 10 min.

### *Gli3 *expression in ICTEV rat models and normal control rats

Real-time PCR (RT-PCR) was performed to evaluate differences in RNA expression levels. The ICTEV model rats were dissected at GD15, GD17, GD19 and GD21. Five embryos with clubfoot were selected and the hindlimbs of the embryos were stored at -70°C. We also dissected control rats at GD15, GD17, GD19 and GD21 and five embryos were selected and their hindlimbs were similarly stored. RNA was extracted from the hindlimbs using a Tissue RNA kit (Tiangen, China) and cDNA was synthesized using a reverse transcription kit (Promega, USA) according to the manufacturer's instructions. Real-time PCR amplification was performed in a 25 μl reaction mixture, which included 12.5 μl SYBR Premix Ex Taq, 9.5 μl deionized water, 0.5 μl (initial concentration 10 μM) of each primer and 2 μl cDNA, according to the manufacturer's instructions (Takara Biotechnology). Amplification was performed by one round of pre-denaturation at 95°C for 10 s, a step-cycle mode of 40 rounds of denaturation at 95°C for 5 s and an annealing and extension step at 58°C for 20 s. The primers used for *Gli3 *were: forward: 5'-TTT TCC AAC ACA GAG GCC TAT TC-3' and reverse: 5'-ATG CAC AAC AAG AGG TCC AAG AT-3'. The primers of β-actin were: forward: 5'-TCC TTC CTG GGT ATG GAA TC- 3' and reverse: 5'-GCA CTG TGT TGG CAT AGA GG-3'.

To evaluate Gli3 protein expression level, we performed western blot analyses. Cytoplasmic protein was extracted from the embryonic hindlimbs of five rats presenting with clubfoot and normal control rat embryonic hindlimbs at GD15 and GD17 using a cytoplasmic and nuclear protein extract kit (Activ Motif, USA) according to the manufacturer's instructions. Protein concentration was determined spectrophotometrically (Unico, USA) at 280 nm[32]. Sample buffer (Beyotime, China) was added to the cytoplasmic protein and the solution was loaded onto a 6% polyacrylamide gel. Following protein separation, the polyacrylamide gel was electro-blotted onto a PVDF membrane (Millipore, MA, USA). Non-specific binding sites were blocked by soaking the membrane in a 3% BSA (Sigma, Poole, Dorset, UK) in TBST buffer (20 mM Tris-buffered saline, 0.047% Tween at pH 7.4) blocking solution overnight at 4°C. The membrane was incubated for 3 hours with rabbit anti-human Gli3 polyclonal antibody (Santa Cruz, USA, 1:50 dilution) at room temperature and washed for 40 min in TBST buffer. The membrane was then incubated for 2 hours at room temperature with a goat anti-rabbit IgG horseradish peroxidase-conjugated antibody (Antibodies Incorporated, USA. 1:4000 dilution). Protein bands were visualized using modified enhanced chemiluminescence (Tiangen, China).

To determine differences in Gli3 levels and localization, we used immunohistochemistry assays. The ICTEV model rats and control rats were dissected at GD19. We selected five embryos presenting with clubfoot from the ICTEV rats and five normal embryos from normal control rats. The hindlimbs of the embryos were embedded in paraffin and fixed in 4% paraformaldehyde overnight at 4°C. The tissues were sectioned into 4-μm slices and labeled with Gli3 antibody (Santa Cruz, USA, 1:50 dilution). The normal control hindlimbs were labeled with Phosphate buffered saline as negative controls. A standard immunohistochemistry protocol was used (Maxim Biotech, Inc., USA).

### *Gli3 *mutation analysis

Genomic DNA was extracted from 84 fresh blood samples obtained from the ICTEV patients using a Blood DNA kit (Tiangen, China) according to the manufacturer's protocol. A previous study investigated mutations in *Gli3 *exons 9, 10, 11 and 12 [26]. We therefore designed primers (shown in Table 1) to amplify the remaining *Gli3 *exons 1-8, 13 and 14 and the 5' flanking sequence. Mutations in *Gli3 *were detected by denaturing gradient gel electrophoresis (DGGE), a sensitive method to separate alleles based on differences in melting behavior [33].

**Table 1:** *Gli3 *primers for DGGE

	Primer sequence	Annealing Temp (°C)	Product size (bp)
Promoter 1 F	*AGCTTGCAGTTCCCTTGC	60	684
R	GTCCGACAATTTCTAACATCGA		
Promoter 2 F	*GCCTTCGATGTTAGAAATTGTCG	62	668
R	TGGGCTGCTGGTAATCCCTGTGC		
Exon 1 F	*TTTTGGAAAGTTGATGGCTCT	60	229
R	GGCTGCTGGTAATCCCTGT		
Exon 2 F	*AATTGCTCCTTAAAGTAGTT	60	335
R	CATAGCTCCTGAACAAGTG		
Exon 3 F	*CAATGTTGCTTTGTGAAT	60	234
R	TAAAAGCCAGCATCTCGT		
Exon 4 F	*CCCCTTGTATCTGGTTTT	57	316
R	GTCTACTTTATACACGTCCC		
Exon5 F	*ATTGCTGATGTGGGTTGT	60	297
R	GTTGCCTTTGCCATTTCC		
Exon 6 F	*TAGGCAAGTAGCAATAAATAG	60	303
R	ACATAATGGATTCAGGAAAA		
Exon 7 F	*GCTCAGCGTTTAAGTGAT	61	268
R	GCATCGACCTGTCCCTCT		
Exon 8 F	*GGGATTGGAGAATTATCAG	60	299
R	AGTCTTGGGAGGAGTGGG		
Exon13 F	*TTCCTTTCCACTTGACCCC	60	391
R	AAAACCCTGAGCAGATGCA		
Exon 14-1 F	*TGTGAGGCAGGCAATGTG	53	325
R	GGAGAAGCAGGGCGAGAT		
Exon 14-2 F	*ATCTCGCCCTGCTTCTCC	55	489
R	GCTGCTGAGGCTGCTGAA		
Exon 14-3 F	*AGAGGATGAGCCTGAAGACG	56	486
R	GCTGCTCGTACCCTGCTT		
Exon 14-4 F	*CTGATGCCAACCTGAACG	56	479
R	TCCACTGGTGCCACTTCC		
Exon 14-5 F	*TGGTCGTCCACCCGCAGAA	58	490
R	GGCCCTTGGTAGATGTTGATGT		
Exon 14-6 F	*AGATGCTTGGGCAGATTAG	60	350
R	TGAACCAGCTTTCGTGTC		
Exon 14-7 F	*CGCTGTGCTCTAATCTGC	60	491
R	TATTGATTTCCGTTGGTTG		

*GC clamp was added at the 5' end of the forward primers

GC clamp: cgcccgccgcgccccgcgcccggcccgccgcccccgcccg

### Identification of the rat *Gli3 *promoter

To obtain a candidate rat *Gli3 *promoter sequence, four forward primers were designed upstream of the rat *Gli3*: Gli3(-1107), 5'-GTG ACC TGC CTG TGC CTG TA-3'; Gli3(-532), 5'-TTA ACC TCT GCG TTA CAA CC-3'; Gli3(-388), 5'-ATC AGA GGG TCT CAG CGT TAG-3'; and Gli3(-128), 5'-CTC CTC AGG CAG AAG ATG CA -3'. Four products were obtained when rat genomic DNA was amplified with each of these primers and the Gli3 reverse primer 5'-ACA CCA CAG TGC CAT CAA A -3'. The amplified fragments were verified by sequencing and cloned into a PMD-18T vector. The clones were digested with *Kpn*I and *Hind*III and cloned into the *Kpn*I and *Hind*III sites of the pGL3-luciferase vector (Promega, USA).

L6GNR4 rat myoblastocytes (Cell Laboratory, Chinese Academy of Sciences, Shanghai) were cultured in Dulbecco's Modified Eagle's Medium supplemented with 10% fetal calf serum, 100 units/mL penicillin, 100 μg/mL streptomycin and then incubated in 5% CO_2 _at 37°C. L6 cells were transfected using a liposome transfection kit (Invitrogen, USA). In each transfection, 12 μg reporter plasmid and 0.1 μg pRL-TK (internal control for transfection efficiency) were used per 6-cm dish. Cells were incubated for 48 hours at 37°C. After transfection, the cells were washed with PBS, lysed with 1× passive lysis buffer and assayed for luciferase activity using the Dual-Luciferase Reporter Assay System (Promega, USA).

### Analysis of the rat *Gli3 *5' region

To search for regulatory elements in the *Gli3 *transcriptional control regions, we analyzed the genomic sequence 1000 bp upstream of the transcription start site using P-Match http://www.gene-regulation.com.

### Chromatin immunoprecipitation assays (ChIP)

ChIP assays were performed according to the manufacturer's instructions (Active Motif, USA). Hindlimbs of GD14 rats were homogenized in PBS on ice. Hoxd13 antibody (8 μg) (Santa Cruz, USA) was used for the immunoprecipitation, and 8 μg of the Sox9 antibody (Santa Cruz, USA) was used as a negative control. DNA was extracted as recommended by protocol. We used 2.5 μL of each sample as a template for PCR using the following primers:

*Gli3 Site1*: ACACTGAGGGCCCTGGGTAG

*Gli3 Site1 rev*: GTGCCGAAAGGGTTGTAACG

*Gli3 Site2*: AACCCTTTCGGCACACTTCTG

*Gli3 Site2 rev*: GTGGCTCTCAACCTTCCTAACG

PCR reactions had an initial denaturing step at 94°C for 5 minutes, followed by 35 cycles of denaturing at 94°C for 35 sec, annealing at 59°C for 35 sec and extension at 72°C for 35 sec, and ended with a final extension at 72°C for 10 min.

### Electrophoretic mobility shift assays (EMSA)

Nucleoprotein was extracted from E14.5 rat embryo limbs using a cytoplasmic and nuclear protein extract kit (Activ Motif, USA) according to the manufacturer's instructions Oligonucleotides and their complementary strands were used to evaluate binding of HoxD13 to the *Gli3 *HoxD13-binding site (5'-*CTAGG*-3'). The double-stranded wild-type (tttaca*CTAGG*attcataaccatagcataattacagcta) and mutated (tttaca*GCTAA*attcataaccatagcataattacagcta) sequences were labeled with biotin according to standard protocols (Pierce, USA). A 100-fold excess of unlabeled probe was used as a specific competitor. The DNA-binding ability of the different proteins was monitored by EMSA on a 10% non-denaturing polyacrylamide gel. DNA binding bands were detected using a chemiluminescence system (Pierce, USA).

## Results

### *Gli3 *is not expressed in the flexor hallucis longus of ICTEV patients

**Figure 1:**
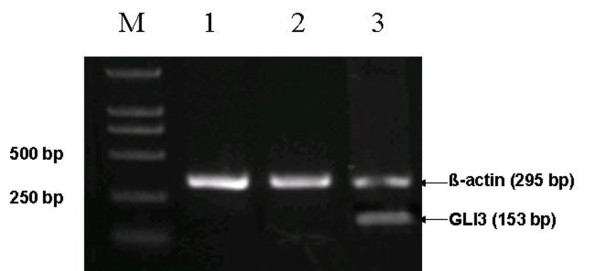
**Semi-quantitative RT-PCR analysis of *Gli3 *mRNA expression in flexor hallucis longus and lung tissue**. *Gli3 *expression was not found in the flexor hallucis longus. Lanes 1 is an RT-PCR sample from an ICTEV patient flexor hallucis longus, lane 2 is a normal control flexor hallucis longus RT-PCR and lane 3 is a normal control lung RT-PCR. *β-actin *expression is shown as a control for all samples. Lane M is a size marker.

To investigate the expression profiles of *Gli3 *in flexor hallucis longus, Semi-quantitative RT-PCR was performed using total RNA from the flexor hallucis longus of patients and cadavers as well as the lung tissues of cadavers. No *Gli3 *expression was detected in the flexor hallucis longus; however, expression was detected in control lung tissue (Fig. 1).

### Identification of rat ICTEV model

**Figure 2:**
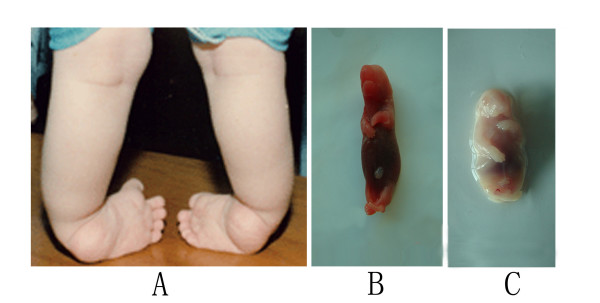
**Comparison of ICTEV and normal embryos at GD21**. (A) Foot of an ICTEV patient, showing cavus, adductus, varus and equines. (B) The hindlimbs of normal embryos at GD21 presented with normal development. (C) The hindlimbs of ICTEV embryos at GD21 presented with dysplasia of the foot, showing cavus and adductus as observed in ICTEV patients.

The embryos were harvested from ATRA-treated pregnant rats at GD15, 17, 19 and 21. We found embryos with clubfoot at GD15. A comparison between ICTEV and normal embryos at GD 21 is shown in Fig 2. Hind limb abnormality was observed in embryos of the ICTEV model rat, similar to that seen in ICTEV patients. A total of 74 embryos with clubfoot were found out of 245 embryos (30.2%), indicating that the ICTEV model was successfully established.

### ICTEV association with *Gli3 *is not due to genetic mutations

Unlike other disorders associated with *Gli3*, no mutations were observed in the 5' flanking sequence or in *Gli3 *exons 1-8, 13 and 14 in the ICTEV patients.

### Gli3 has higher expression in ICTEV model rats than in control rats

**Figure 3:**
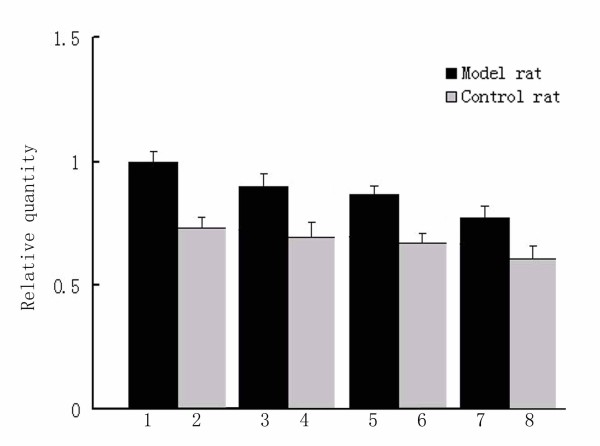
**Real-time PCR analysis of *Gli3 *expression in the hindlimb of ICTEV model rat and normal control rat embryos**. Compared to the normal control, *Gli3 *mRNA expression was significantly enhanced. With birth drawing near, *Gli3 *expression in both model and normal rats had the tendency to decrease. Column 1, 3, 5 and 7 show *Gli3 *relative expression in ICTEV model rat at GD15, GD17, GD19 and GD21, respectively. Column 2, 4, 6 and 8 show *Gli3 *relative expression in normal control rat embryos at GD15, GD17, GD19 and GD21, respectively.

**Figure 4:**
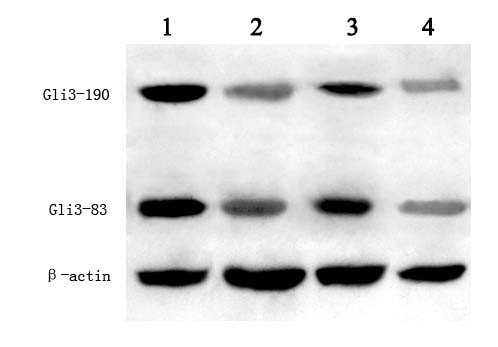
**Western blot results**. Western blots were used to determine Gli3 protein abundance in nuclear extracts from the hindlimb tissue of ICTEV model rat and normal control rat embryos. Compared to the normal control, *Gli3 *expression was significantly enhanced in model rats. With birth drawing near, *Gli3 *protein expression in both model and normal rats had a tendency to decrease. Lane 1 is the protein of ICTEV model rat embryos at GD15, lane 2 is the protein of normal rat embryos at GD15, lane 3 is the protein of ICTEV model rat embryos at GD17 and lane 4 is the protein of normal rat embryos at GD17.

**Figure 5:**
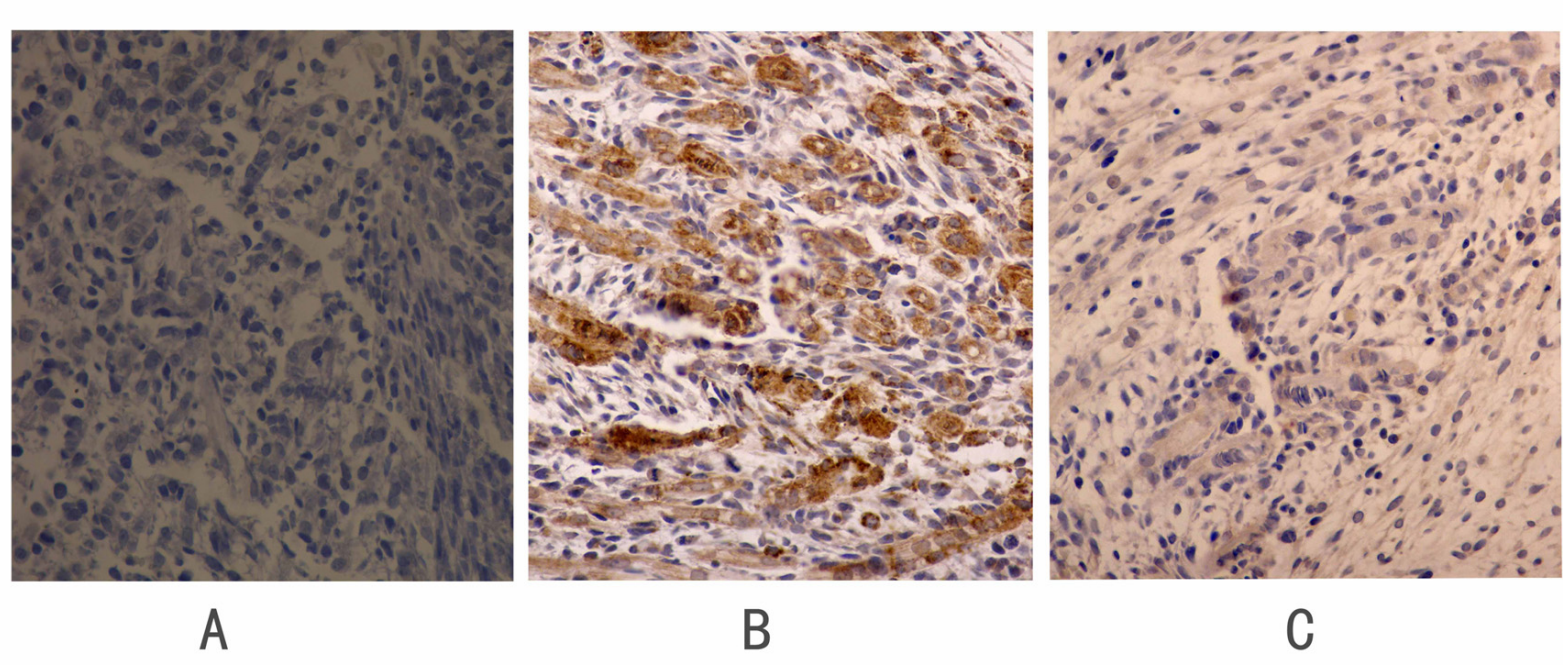
**Immunohistochemistry results**. Immunohistochemistry revealed that Gli3 expression in the hindlimb tissue of ICTEV model rat embryos was higher than that in normal control rat embryos at GD19 (400×). (A) Hindlimb tissue of a normal control rat embryo labeled with PBS. (B) Hindlimb tissue of an ICTEV model rat embryo labeled with Gli3 antibody. (C) Hindlimb tissue of a normal control rat embryo labeled with Gli3 antibody.

To study *Gli3 *earlier in development, we used a rat model for ICTEV. Expression of *Gli3 *was higher in ICTEV model rat embryonic hindlimbs compared to normal control rat embryonic hindlimbs (Fig. 3). Similarly, Gli3 protein showed increased abundance in ICTEV model rat embryonic hindlimbs compared to normal control rat embryos (Fig. 4). For both ICTEV model rats and normal control rats, *Gli3 *RNA levels and Gli3 protein abundance decreased during embryonic development (Figs. 3 and 4). Using immunohistochemistry, the Gli3 protein in ICTEV rats was localized to the foot muscle of the rats (Fig. 5).

### *Gli3 *promoter identification

**Figure 6:**
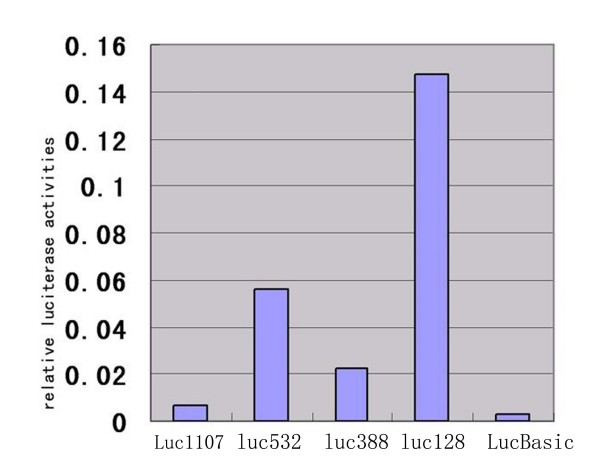
**Relative activities of rat Gli3 promoter regions in a luciferase reporter construct**. Activities were measured in L6 cells. Findings suggested positive regulatory elements existed in the regions from -1107 to -532, -388 to -128 and from -128 to -48 upstream of *Gli3 *and negative regulatory elements in the region from -532 to -388 upstream of *Gli3*.

To determine the promoter region of *Gli3*, we generated four luciferase reporter constructs (Luc1107, Luc532, Luc388 and Luc128) with fragments upstream of *Gli3 *as the promoters. All the luciferase reporter constructs showed higher promoter activity compared to the pGL3-basic reporter vector. Relative to the pGL3-basic reporter vector, Luc128, Luc388, Luc532 and Luc1107 increased luciferase activity 2-fold, 20-fold, 8-fold and 53-fold, respectively (Fig. 6). These data suggest that there are positive regulatory elements in the regions from -1107 to -532, -388 to -128 and from -128 to -48 upstream of *Gli3 *and negative regulatory elements in the region from -532 to -388 upstream of *Gli3*.

### HoxD13 binding sites in the 5' region of the rat *Gli3*

**Figure 7:**
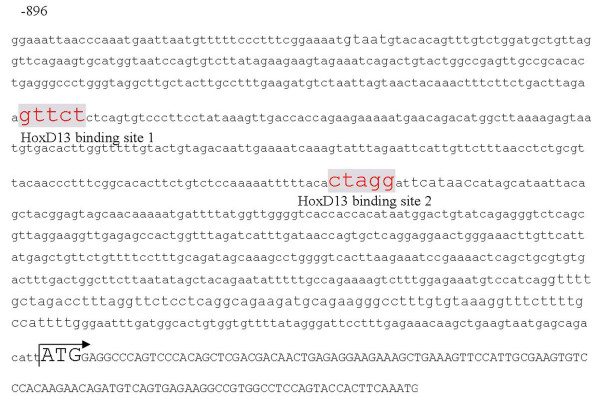
**Potential binding sites**. The rat *Gli3 *proximal promoter region contains two potential HoxD13 binding sites (gray boxes). The transcription start site is indicated by an arrow. The *Gli3 *protein translation start site (ATG) is indicated.

To identify the transcription factors that directly regulate *Gli3 *expression, we analyzed the genomic region upstream of the transcription start site of the *Gli3 *gene with the transcription binding-site prediction program P-Match. Two putative HoxD13 binding sites, the HoxD13 binding site 1 (-667): ***gttct ***and the HoxD13 binding site 2 (-477):***ctagg ***were identified, suggesting that Hoxd13 could directly bind to these sites to regulate *Gli3 *expression (Fig. 7).

**Figure 8:**
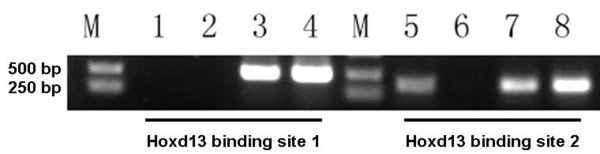
**ChIP results**. The ChIP assay of putative HoxD13 binding sites 1 and 2 in the *Gli3 *promoter. Only HoxD13 binding site 2 binds HoxD13 *in vivo*. In lanes 1 and 5, chromatin from rat embryonic hindlimbs was immunoprecipitated with the HoxD13 antibody. In lanes 2 and 6, the Sox9 antibody was used as a negative control. Lanes 3 and 7 show the enzymatic shearing before immunoprecipitation. Genomic DNA was used as a positive control (lanes 4 and 8). Lane M is a size marker.

**Figure 9:**
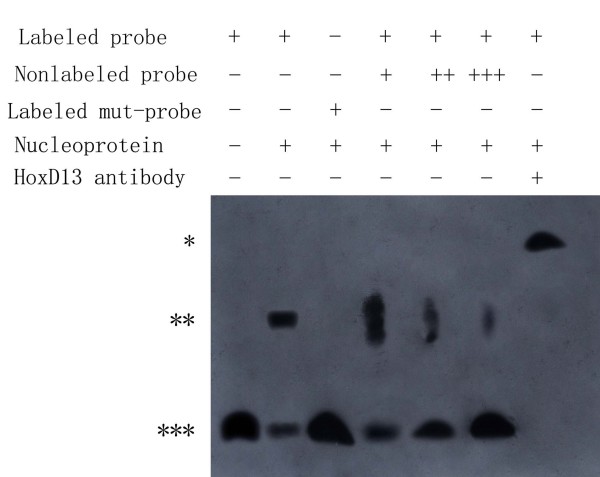
**EMSA results**. Electrophoretic mobility shift assay (EMSA) using oligonucleotide probes containing the HoxD13 binding site 2. The *** denotes unbound biotin-labeled DNA, ** denotes Hoxd13-DNA complexes and * denotes Hoxd13-antibody-Hoxd13-DNA complexes.

To verify the *in vivo *binding of Hoxd13 to the *Gli3 *promoter, we used ChIP. Cross-linked and sheared chromatin from rat GD14 embryonic hindlimbs were immunoprecipitated with an anti-Hoxd13 antibody and analyzed by PCR. These ChIP analyses revealed that, *in vivo*, Hoxd13 efficiently bound only to binding site 2 (Fig. 8). Additionally, nucleoprotein containing Hoxd13 was incubated with a fragment of -483 ~ -446 bp upstream of Gli3 containing site 2. As shown in Fig. 9, strong DNA binding was observed in the presence of the Hoxd13 protein. A competition experiment and supershift existence in the presence of the Hoxd13 antibody demonstrated the specificity of such binding. The result indicated Hoxd13 is bound to site 2 in vitro.

## Discussion

The mammalian *Gli *gene family encodes zinc finger transcription factors and plays a role in developmental regulation and human diseases [34]. One member of this family, *Gli3*, was identified as a candidate gene for ICTEV [26]. To investigate the mechanism of this association, we investigated Gli3 mRNA and protein expression patterns in ICTEV patients. *Gli3 *was not expressed in the flexor hallucis longus of ICTEV patients, suggesting that although *Gli3 *is important for correct limb development, it is no longer expressed in postnatal limbs.

To investigate the role of *Gli3 *role in ICTEV earlier in development, we established an ICTEV rat model and detected *Gli3 *expression in rat embryonic hindlimbs. There are some concerns regarding the rat model of ICTEV induced with ATRA in pregnant rats. The rat model has some limitations. After induction with ATRA, not all fetal rats were exhibited with symptoms of ICTEV. Those that developed the clubfoot also developed malformations such as rima oculi, spina bifida, cranial deformation and anal atresia. Although the rat model of ICTEV induced with ATRA is not ideal and is controversial, it is currently the best rat model of ICTEV. We tested different dosages of ATRA in preliminary experiments and found that 135 mg/kg was the dose at which the highest percentage (about 30%) of clubfeet in the lower limbs was achieved. We only selected the fetal rats with clubfeet as the experimental subjects to ensure the most legitimate comparison to patients with ICTEV. We found that both Gli3 mRNA and protein expression levels were higher in the ICTEV model rats compared to normal control rats. Furthermore, with birth drawing near, the *Gli3 *gene expression in both of the normal control group and the model group was gradually downregulated (Figs. 3 and 4), indicating that the *Gli3 *gene is a very important gene in regulating the limb development and that its role of regulating limb development may disappear after birth. This is also consistent the inability to detect the *Gli3 *gene in the flexor hallucis longus of adults (Fig. 1), which may be because it is not expressed in the lower limb post-birth. The occurrence of ICTEV may be caused by changes in the expression level of this critical gene that is related to limb development. Immunohistochemical results suggest that *Gli3 *protein expression in the muscles around ankles of model fetal rats was remarkably higher than in the control group, and the affected sites of patients with ICTEV were also in the ankle. The location of this muscle was identical to that of flexor hallucis longus; therefore, it is reasonable to conclude that the occurrence of ICTEV is associated with changes in the expression level of *Gli3*. To identify the underlying reason for the change in *Gli3 *expression, we first looked for mutations in the *GLI3 *promoter and coding regions of ICTEV patients. A previous study investigating mutations in *GLI3 *exons 9, 10, 11 and 12 identified only one polymorphism [26]. To complete this study, we sequenced the remaining exons and the promotor, but did not detect any polymorphisms. While a larger sample size might reveal polymorphisms, these data suggest that the *Gli3 *mutation is not the root of its association with ICTEV.

The changes in *Gli3 *gene expression may be caused by changes in some transcription factors that regulate the *GLI3 *gene, ultimately leading to the development of ICTEV. Accordingly, future studies should focus on the regulation of the *GLI3 *gene and its function.

Alternatively, *Gli3 *could be differentially regulated in ICTEV model rat embryos compared to normal control embryos. To determine the transcription factors that bind to the promoter region of *Gli3*, we constructed a series of rat *Gli3 *truncated promoters and cloned them into reporter gene constructs. These analyses suggested that the promoter has both positive and negative regulatory elements. Putative HoxD13 binding sites 1 and 2 in *Gli3*'s upstream 5' region were located in positive and negative regulatory elements, respectively. HoxD13, however, only bound to site 2 *in vivo*. *In vitro *competition experiments also revealed a specific affinity of Hoxd13 to this site. Thus, Hoxd13 can directly regulate the expression of *Gli3 *during limb formation. We cannot, however, exclude the possibility that Hoxd13 interacts with the *Gli3 *promoter regions as a part of a protein complex. In ICTEV patients, *HOXD13 *has lower expression compared to healthy subjects [27]. Thus, the decrease in *HOXD13 *expression maybe led to a change in the expression of *GLI3*, which manifests as ICTEV.

This study provides a theoretical basis for the pathogenesis of ICTEV. Future studies will investigate how *HoxD13 *regulates *Gli3 *during limb development and will provide a clearer mechanism for the pathogenesis of ICTEV.

## Conclusion

Our study indicates that HoxD13 directly interacts with the promoter of *Gli3 *and Gli3 mRNA and protein expression levels were increased in the ICTEV model rats. These findings suggest that HoxD13 is a transcription factor of *Gli3*. Low expression of *HOXD13 *might lead to increase GLI3 expression level during limb formation, which likely plays a key role in ICTEV pathogenesis.

## Competing interests

The authors declare that they have no competing interests.

## Authors' contributions

DHC is the guarantor of the study. He designed the study and was the primary writer of the manuscript. MHR and CKL designed and wrote the study and critically revised the study for its content. XZ and NZ initiated and monitored the study. CLJ supervised and critically revised the study for its content. All authors read and approved the final manuscript.

## Pre-publication history

The pre-publication history for this paper can be accessed here:

http://www.biomedcentral.com/1471-2474/10/142/prepub
